# The Role of Nutrients in Reducing the Risk for Noncommunicable Diseases during Aging

**DOI:** 10.3390/nu11010085

**Published:** 2019-01-04

**Authors:** Maaike J. Bruins, Peter Van Dael, Manfred Eggersdorfer

**Affiliations:** 1 Nutrition Science & Advocacy, DSM Nutritional Products, CH-4303 Kaiseraugst, Switzerland; peter.van-dael@dsm.com; 2 University Medical Center Groningen, 9713 GZ Groningen, The Netherlands; m.l.eggersdorfer@rug.nl

**Keywords:** chronic disease, noncommunicable disease, nutrient inadequacies and deficiencies, nutrient interventions, public health, musculoskeletal disorders, dementia, eye disorders, cardiovascular disease

## Abstract

An increasing aging population worldwide accounts for a growing share of noncommunicable diseases (NCDs) of the overall social and economic burden. Dietary and nutritional approaches are of paramount importance in the management of NCDs. As a result, nutrition programs are increasingly integrated into public health policies. At present, programs aimed at reducing the burden of NCDs have focused mostly on the excess of unhealthy nutrient intakes whereas the importance of optimizing adequate essential and semi-essential nutrient intakes and nutrient-rich diets has received less attention. Surveys indicate that nutrient intakes of the aging population are insufficient to optimally support healthy aging. Vitamin and mineral deficiencies in older adults are related to increased risk of NCDs including fatigue, cardiovascular disease, and cognitive and neuromuscular function impairments. Reviewed literature demonstrates that improving intake for certain nutrients may be important in reducing progress of NCDs such as musculoskeletal disorders, dementia, loss of vision, and cardiometabolic diseases during aging. Current knowledge concerning improving individual nutrient intakes to reduce progression of chronic disease is still emerging with varying effect sizes and levels of evidence. Most pronounced benefits of nutrients were found in participants who had low nutrient intake or status at baseline or who had increased genetic and metabolic needs for that nutrient. Authorities should implement ways to optimize essential nutrient intake as an integral part of their strategies to address NCDs.

## 1. Introduction

Globally, significant gains in human longevity have been made in the last couple of decades as evidenced by an average 5.5-year increase in life expectancy between 2000 and 2016 [1]. In many countries average life expectancy currently exceeds 80 years [1]. These longevity gains have come at a cost, however, with the most obvious being an increase in age-related diseases [2]. Noncommunicable diseases (NCDs) such as diabetes, musculoskeletal disorders, cardiovascular diseases, neurological disorders, and cancers increase with age, and place a burden on individuals and healthcare systems [3]. Supporting healthy aging by preventing NCDs is a major priority for agencies such as the World Health Organization (WHO) and United Nations [4,5]. 

The WHO estimates that NCDs contribute 1.6 billion disability-adjusted life-years (DALYs) to the global burden of disease and identified unhealthy diets and physical inactivity are among the main modifiable risk factors, together with excess alcohol and tobacco use [6]. Nutrition is an important determinant of human health by providing the essential building blocks for growth, development, and maintenance of a healthy status throughout life [7,8]. In this context, the co-existing burdens of undernutrition and overnutrition represent a paradigm shift for health authorities requiring appropriate dietary management recommendations [9]. Modern lifestyles and easy access to high-energy, low-nutrient rich foods are considered part of the problem [3,10,11,12]. For example, the economic costs of unhealthy diets and low physical activity in the EU were calculated to be €1.3 billion per year [13]. 

Currently, health authorities mainly target problems associated with obesity and cardiovascular diseases by focusing on reducing excess intake of calories, sugar, salt, and saturated fats. However, the importance of a positive message associated with promoting adequate nutrient intake as part of a balanced diet should not be overlooked [4]. There is considerable variation in the consumption of food items that need to be encouraged and food items which should be limited, both between and within different countries. This was reflected in a recent study in European countries showing suboptimal nutrient-density of diets and significant proportions of the population consuming excess amounts of salt, sugar and saturated fat, as well as significant proportions of the population not meeting the required or adequate intakes for various essential nutrients (Table 1) [12].

The health consequences of poor nutrition almost certainly accumulate over the lifespan of the individual. Table 2 presents information regarding some of the more frequently reported chronic clinical signs associated with certain vitamin and mineral deficiencies in older adults. Clinical signs and symptoms are mostly nonspecific and difficult to diagnose. During the aging process, a number of changes occur, such as increased medication use, reduced food intake due to lower food appeal, and compromised nutrient absorption. These complex changes prevent elderly persons from meeting their nutritional requirements. This consequently leads to increased risk of malnutrition, frailty, and reduced quality of life (QoL) [14,15,16,17].

Health policies and interventions to improve dietary intake at the population level are essential to reverse the global trend towards unhealthy dietary patterns and physical inactivity. However, more individualized approaches may be needed to address persistent nutritional gaps and prevent future morbidity in high-risk groups such as the older population [19,20]. An estimated 5% to 10% of community-dwelling adults >70 years of age are undernourished; this proportion rises to 30% to 65% among institutionalized elderly patients. In the older adult population, nutrients of concern include, among others, calcium, vitamin D, and vitamin B_6_ and B_12_ [15,20,21]. Vitamin D deficiency was found not only to be a problem in the elderly, but to be a global problem common across all age ranges [22]. Genetic variations play a role in dietary response and genetic variations also play a role in determining nutrient status and requirements [23]. By understanding the genome that affects the individual requirements for and response to nutrition, diseases of aging that have a nutritional component can be addressed in a targeted way.

In general, activities endorsing lifestyles that include healthy diets have usually focused on limiting the consumption of salt, sugar, and saturated fat. However, focus on the need to meet adequate dietary intake of essential nutrients through a healthy diet is considered equally important.

This review focuses on the role of nutrients in the risk reduction of NCDs in disorders prevalent in the aging population and for which the societal costs are substantial [24]. The evidence for a connection between NCDs and inadequate intake or status of specific nutrients such as vitamins, carotenoids, omega-3 fatty acids, and other bioactive substances is reviewed. Furthermore, the impact of interventions aimed at correcting these inadequacies will be discussed.

## 2. Musculoskeletal Health in the Older Adult

The gradual loss of bone mass and disruption of bone architecture associated with osteoporosis results in an increased risk of bone fractures, particularly of the hip, spine, and wrist. It is an age-related chronic, complex, multifactorial skeletal disorder which affects both men and women, particularly postmenopausal women [25]. Osteoporosis places a huge personal and economic burden on society. In Europe, for example, the disability caused by the disease is greater than that caused by cancers (with the exception of lung cancer) and is comparable or greater than that caused by a variety of chronic NCDs, such as rheumatoid arthritis, asthma and hypertension-related heart disease [26].

In a WHO report it was noted that the remaining lifetime risk of an osteoporotic fracture in women aged 50 years in developed countries was >40% (>20% for hip fracture) [27]. At the time of this report, osteoporotic fractures had the sixth highest disease burden in the Americas and Europe combined, as estimated by disability-adjusted life years [27,28]. In 27 countries in the European Union, based upon the overall epidemiology of 22 million women and 5.5 million men with osteoporosis, it was calculated that this would result in 3.5 million new bone fractures (hip, 610,000; vertebral, 520,000; forearm, 560,000; and others 1.8 million) [28]. The economic burden to manage these incident and prior bone fractures was calculated to be €37 billion.

In the elderly, both micronutrient and macronutrient deficiencies appear to contribute to the pathogenesis of skeletal fractures as a consequence of age-related bone loss and frailty [16]. Nutrients that play a role in bone metabolism include vitamin D and vitamin K, calcium, magnesium, phosphorus, proteins, and fatty acids. 

### 2.1. Vitamin D in Musculoskeletal Health

Vitamin D is involved in bone homeostasis by enhancing calcium and phosphorus absorption from the intestine and maintaining adequate levels in blood. Low vitamin D levels have been mainly implicated in musculoskeletal disorders including bone and muscle health [29]. Serum levels of 25(OH)D have been associated with bone turnover markers levels [30]. 

Vitamin D comprises a group of secosteroids (calciferols), and in humans the two most important compounds in this group are vitamin D_3_ (cholecalciferol) and vitamin D_2_ (ergocalciferol) [22]. A major part of vitamin D comes from UV-B induced production in the skin and only about 20% from dietary intake. Dietary sources are limited to mainly oily fish and foods fortified with the vitamin [31]. Lack of vitamin D from the diet and increased awareness of the harmful skin effects of excessive sunlight exposure have contributed to low vitamin D status and even deficiency globally. 

**Figure 1:**
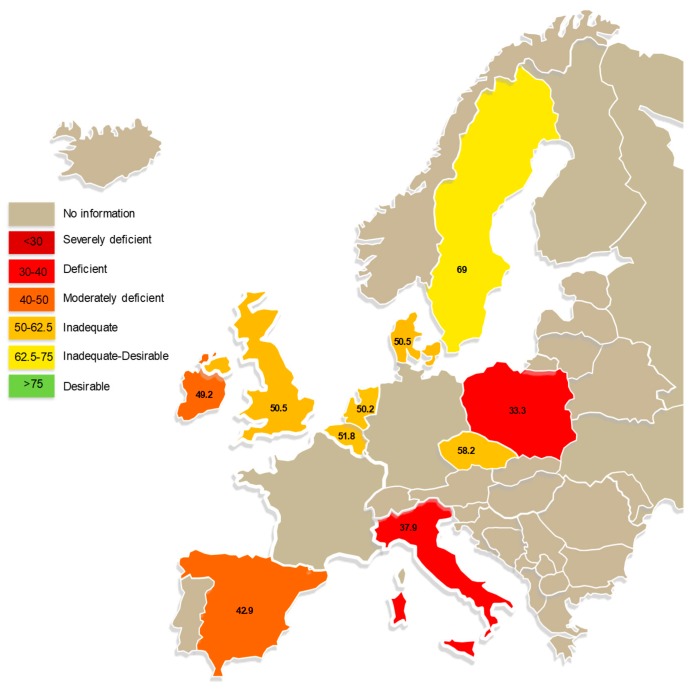
Europe map of vitamin D deficiency in older adults (mean 25(OH)D status (nmol/L) in adults aged ≥50 years) (based on: [39]).

Serum 25-hydroxyvitamin D is the most widely used indicator for vitamin D status in clinical practice and, while 25–50 nmol/L is generally defined as insufficiency with regards to bone health, for optimal calcium absorption and control of secondary hyperparathyroidism a level closer to 75 nmol/L has been proposed [16,22,32,33]. Most researchers agree that 25-hydroxyvitamin D levels below 50 nmol/L are associated with lower bone mineral density [22]. Likewise, the effect of vitamin D deficiency on fracture risk is difficult to quantify, but large population studies found that hip fracture risk was higher in those with a 25-hydroxyvitamin D level below 50–62.5 nmol/L [34,35]. Based on a serum 25-hydroxyvitamin D level of <30 nmol/L it was reported that on average 13% of 55,844 European individuals had moderate or severe vitamin D deficiency, and this increased to 40% of individuals with mild to severe deficiency if a level of <50 nmol/L was included [36]. The authors noted that vitamin D deficiency was present across Europe and was both a clinical and public health concern requiring urgent action. Similar levels of vitamin D deficiency and concern have been reported by many research groups worldwide [22,36,37,38]. Figure 1 highlights the variable levels of vitamin D deficiency across Europe [39].

The role of vitamin D and related analogues, with or without calcium, for preventing bone fractures in post-menopausal women and older men was the subject of a Cochrane review [25]. This systematic review included 53 trials and 91,791 older women or men aged over 65 years from community, hospital, and nursing-home settings, and assessed the impact of vitamin D for the prevention of hip or other types of fracture. In this analysis vitamin D alone did not appear to have a significant effect on fracture prevention, whereas vitamin D in combination with calcium significantly reduced the likelihood of hip fractures (*P* = 0.01), non-vertebral fractures and any type of fracture. Hip fracture incidence was particularly reduced in institutionalized residents with a risk reduction of 25%. In a separate systematic review (30 randomized controlled trials (RCTs) involving 5615 subjects of mean age 61 years), vitamin D supplementation was shown to produce a small but statistically significant improvement in global muscle strength. The most benefit was observed in individuals who presented with a 25-hydroxyvitamin D level below 30 nmol/L (compared with those with a level ≥30 nmol/L), and in subjects aged 65 years or older [29].

The economic value of vitamin D supplementation has been the subject of several health economic evaluations showing that increasing vitamin D status through supplementation or fortification can prevent fractures and improve QoL in older adults and associated health care costs [40,41,42,43,44].

### 2.2. Vitamin K in Musculoskeletal Health

Two forms of vitamin K exist: vitamin K_1_ (phylloquinone, mainly found in green leafy vegetables) and vitamin K_2_ (menaquinone, mainly found in fermented dairy and produced by lactic acid bacteria in the intestine). Vitamin K is required for promoting osteoblast differentiation, upregulating transcription of specific genes in osteoblasts, and activating bone-associated vitamin K dependent proteins, which play critical roles in extracellular bone matrix mineralization. Less is known about vitamin K and health, but there is growing evidence suggesting a synergistic effect between vitamins K and D in bone [40]. A number of studies reported that vitamin K is essential for optimization of bone health with benefits in preventing bone loss [41]. Vitamin K_2_ supplementation combined with vitamin D and calcium for 2 years in a randomized placebo-controlled trial resulted in a significant increase in bone-mineral density and content in older women [42]. In another recent RCT it was found that combined vitamin K_2_, vitamin D and calcium supplementation for 6 months increased the bone mineral density of lumbar 3 spine vertebra compared to vitamin D and calcium alone in postmenopausal Korean women [43].

Current research investigating the effect of vitamin D alone or in combination with other nutrients on fractures, cardiovascular disease, diabetes, cognitive function, immunity, and other benefits is ongoing in two large scale studies in older adults (DO-HEALTH in Europe, FIND in Finland). In addition, many research groups engage in basic science to study the combined action of vitamin K2, vitamin D, and calcium, and their function on the molecular level. More studies are required that target vitamin D supplementation in combination with other nutrients such as calcium and vitamin K where it is needed, in people with vitamin D deficiency or older people, who are more likely to be frail in institutionalized residents.

## 3. Cognitive Disorders

Dementia is a term that describes a decline in cognitive abilities including memory, and reduction in a person’s ability to perform everyday activities [44]. Dementia prevalence is forecast to increase dramatically in future years [45]. At present about 50 million people have dementia worldwide, and this is projected to reach 80 million by 2030 and 150 million by 2050 [46]. Alzheimer’s disease (AD) is the most common form of dementia in people aged >60 years, accounting for 60–70% of the total number of cases and is the major focus of this section [46]. Vascular dementia is the second most common cause of dementia with at least 20% of dementia cases. 

Alzheimer’s disease is a complex, progressive, multifactorial, neurodegenerative disease [24,45]. The presentation generally involves progressive memory loss, impaired thinking, disorientation, and changes in personality and mood. As the disease advances there is a marked reduction in cognitive and physical functioning [47,48]. Genetic factors account for about 70% of the risk contributing to AD, while modifiable factors related to general health and lifestyle may also be involved [48]. Risk factors for vascular dementia are predominantly modifiable and of vascular origin (including hypertension, diabetes mellitus, dyslipidemia, and the metabolic syndrome). Managing non-genetic risk factors effectively may provide opportunity to prevent and treat the progressive cognitive decline associated with AD [47]. The focus of this section of the review is on nutritional status and its potential role in AD. 

### The Role of Nutrition in Dementia

In terms of a link between nutrient status in older adults and cognition, evidence exists for B-vitamins, and vitamin C, D, and E, as well as the omega-3 long chain polyunsaturated fatty acids (LCPUFAs) docosahexaenoic acid (DHA) and eicosapentaenoic acid (EPA), as reviewed by Antal et al. [48] and summarized here (unless otherwise noted). 

Folic acid and vitamin B_6_ and B_12_ are important in the nervous system at all ages, but particularly in elderly people, deficiency contributes to aging brain processes [49]. Low status of folic acid and vitamins B_6_ and B_12_ are among the risk factors for elevated homocysteine. With respect to dementia, there is reasonable evidence linking lower levels of folic acid, vitamin B_6_, vitamin B_12_, and higher concentrations of homocysteine with age-related cognitive decline [50]. One of the mechanisms involved may the impaired methylation processes due to folic acid and vitamin B_12_ deficiency that lead to accumulation of homocysteine affecting mood and some cognitive functions [50]. In several RCTs supplementation with folic acid, vitamin B_12_, and vitamin B_6_ for at least 2 years has been investigated [44]. However, the findings of a recent meta-analysis reported that B vitamins had little to no effect with respect to preventing cognitive decline [51]. Notably, individuals with high homocysteine levels had significant cognitive decline and B-vitamins were found to improve memory only in this subgroup [52]. Also, evidence exists that in elderly subjects with an increased risk of dementia, B-vitamins can slow brain shrinkage over two years by up to 30% [53]. At present, the evidence is insufficiently compelling to support B-vitamin supplementation to prevent cognitive decline and dementia. 

Dehydroascorbic acid, a metabolite of vitamin C, is a potent antioxidant, an essential cofactor in many enzymatic reactions, and has a role in metabolizing cholesterol. Large dietary surveys undertaken in Germany, the Netherlands, the UK, and the US indicated inadequate vitamin C intake in up to half of respective populations [54]. As with vitamin E, however, studies of vitamin C in patients with AD have been equivocal. The overall conclusion of the Team of Alzheimer Drug Discovery Foundation is that maintaining adequate levels of vitamin C through diet may offer more benefit than supplementation. 

The metabolically active form of vitamin D, 1,25-dihydroxy vitamin D, binds to vitamin D receptors that are present in brain regions involved in cognition. Proposed mechanisms for the protective effects of vitamin D against cognitive decline include clearing Aβ peptide, regulating intraneuronal calcium, anti-inflammatory activity, antioxidative activity, preventing and reducing ischemia, and regulating choline acetyltransferase neurotrophic agents. There is strong evidence that patients with AD have lower vitamin D status than healthy controls and that lower vitamin D status is associated with increased risk of developing dementia. Although vitamin D supplementation alone was insufficient to improve cognition in a study of patients with newly diagnosed AD, the Vitamin D Council recommends that middle-aged and older adults maintain vitamin D blood levels in the higher range of normal (175–200 nmol/L; 70–80 ng/mL). 

Vitamin E possesses antioxidant properties which may prevent hyperphosphorylated tau protein dysfunction and has been shown to reduce the rate of Aβ protein-induced death in cultures of hippocampal and cortical cells [55]. The γ-tocopherol isomer is particularly effective in scavenging free radicals that cause inflammation [55]. By scavenging Aβ protein-associated free radicals, vitamin E may have a neuroprotective effect during oxidative stress. However, while promising in principle, studies of α-tocopherol supplementation in patients with AD have not been convincing and dietary vitamin E may provide greater protection against age-related neurodegenerative conditions. 

Brain membranes are composed mainly of phospholipids, predominantly the LCPUFAs DHA and arachidonic acid (ARA). DHA has multiple actions in maintaining neurological function. Lower plasma DHA has been associated with cognitive decline in both healthy elderly people and AD patients [56]. Investigations to date of the therapeutic potential of supplementation or higher dietary intake of DHA in patients with AD have produced conflicting results, although it is possible that cognitive impairment in the study populations was already resistant to intervention. Given the essential role of DHA in the human brain, a general recommendation to maintain an adequate dietary intake of DHA throughout adulthood appears to be a reasonable approach to prevent cognitive decline. 

Due to a high concentration of oxygen free radicals relative to antioxidative defenses in the brain, it may be especially vulnerable to oxidative stress and consequent damage to lipids and proteins [57]. AD is also associated with lower levels of acetylcholine in the hippocampal and cortical regions, resulting in memory impairment. Fruits, vegetables, coffee, and cereal grains contain high levels of polyphenols. In vitro and animal studies of specific dietary flavonoids and plant extracts have shown reduction of oxidative stress and inhibition of acetylcholinesterase, suggesting a dual protective role for polyphenols against cognitive decline and dementia [57]. Although no conclusions can be drawn about the relative benefits of any particular plant polyphenol over another, the findings emphasize the importance of life-long consumption of foods with high content of these antioxidants. 

Trials that have reported no effect of nutrients generally included older adults who were unlikely to have a marked decline in cognitive function [52]. Trial design should consider including older individuals with deficiencies that increases their risk of cognitive decline, and who may benefit from nutrition intervention. Sensitive assessment tools and surrogate markers are needed that examine specific aspects of brain structure and function such as neuroimaging techniques to advance the understanding of nutrition interventions that could reduce the risk of dementia. 

## 4. Eye Disorders

Impairments of the essential senses of vision and hearing are the second-leading cause of years of lived with disability [58]. The most common causes of vision loss among the elderly are age-related macular degeneration, glaucoma, cataracts, and diabetic retinopathy [59]. Aging is the greatest risk factor associated with the development of age-related macular degeneration, but also environmental and lifestyle factors such as smoking, oxidative stress, and diet may significantly affect the risk [60]. Recent studies suggest that increasing exposure to blue light emitted by electronics and energy-efficient lightbulbs over time could lead to damaged retinal cells which on the long-term can cause vision problems like age-related macular degeneration [61]. Eye health problems in the ever-increasing aging generation, and “exposure to blue light” may result in a new NCD. 

Carotenoids have a range of functions in human health and, in particular, there is evidence that they have beneficial effects on eye health [62]. Two dietary carotenoids, lutein and zeaxanthin are macular pigments found in the human retina [63]. Macular pigment has local antioxidant properties and absorbs high energy, short wavelength blue light protecting the retina from photochemical damage [64]. Macular pigment can neutralize ROS, protect against UV-induced peroxidation, and reduce the formation of lipofuscin and associated oxidative-stress induced damage [63]. Thus, the carotenoids provide potential benefits for ocular function and health. 

Individuals who have low macular pigment optical density levels (0.2 or lower) may benefit from supplementation with lutein/zeaxanthin which can help increase macular pigment optical density levels [65,66,67,68,69,70,71,72]. For retinal protection, macular pigment optical density values of 0.4 to 0.6 are desirable, especially in older adults [73]. Dietary intake of lutein and zeaxanthin may differ with age, sex, and ethnicity. Across all age groups the intake of lutein is higher than for zeaxanthin and this is independent of sex and ethnicity. In addition, lower zeaxanthin to lutein ratios are reported for groups at risk of age-related macular degeneration (e.g., the elderly and females) [74]. A number of studies, including some in healthy subjects, have demonstrated that lutein/zeaxanthin supplementation can improve visual performance, including contrast sensitivity, glare tolerance and photo stress recovery [65,66,67,68,69,70,71,72,75,76].

Age-related macular degeneration is an increasing problem among the elderly and studies of the effects of lutein/zeaxanthin supplementation have produced mixed results. However, important data were provided by secondary analyses of the large Age-Related Eye Disease Study 2 (AREDS2) [77,78]. This randomized trial investigated the effect of adding lutein/zeaxanthin 10/2 mg, DHA (350 mg) + EPA (650 mg), or both to the original AREDS2 formulation (vitamin C, vitamin E, β-carotene, zinc, and copper) or to variations of this formulation (excluding β-carotene and/or with reduced zinc). Participants (*n* = 4203) were followed for a median 5 years. The primary analysis found no additional beneficial or harmful effect for lutein/zeaxanthin and/or omega-3 fatty acids on progression to late age-related macular degeneration compared with the original AREDS1 formula using β-carotene instead of lutein/zeaxanthin. However, a prespecified secondary analysis found a significant 26% risk reduction for progression to advanced age-related macular degeneration when comparing lutein/zeaxanthin supplementation with no lutein/zeaxanthin supplementation in the quintile with the lowest dietary intake of these two carotenoids (median 0.7 mg/day), as indicated by a hazard ratio of 0.74 (95% confidence interval 0.59–0.94, *p* = 0.01). In addition, a post hoc analysis showed that lutein/zeaxanthin (excluding β-carotene) was more effective than the original AREDS formulation containing β-carotene but no lutein/zeaxanthin for reducing progression to advanced age-related macular degeneration (hazard ratio 0.82, 95% CI 0.69–0.96, *p* = 0.02) [77].

There is also some evidence suggesting there is a relationship between lutein/zeaxanthin status and the risk of developing nuclear cataracts [79], and in the AREDS2 trial the addition of lutein/zeaxanthin supplementation reduced the risk of cataract surgery in the quintile with the lowest dietary intake of these carotenoids (hazard ratio 0.68, 95% CI 0.48–0.96, *p* = 0.03) [80]. 

If the AREDS2 complex (i.e., vitamin C and E, zinc, copper, lutein/zeaxanthin and omega-3 fatty acids) was used by all adults aged >55 years, it has been estimated this would result in an average of about 1 million avoided age-related macular degeneration and cataract events per year in the USA (based on a risk reduction of 23.6% for age-related macular degeneration and 16.2% for cataracts). This would result in a net annual cost saving of US$1.2 billion, mostly as a consequence of reduced healthcare expenditure [81]. Establishing intake recommendations for lutein is an important step forward to support optimal visual performance and reduce the risk of age-related macular eye disease in the general population. This would be a relevant contribution to public health in the face of a globally aging population.

Future studies may include additional assessments of the relationship between macular pigment and different genotypic and phenotypic forms of age-related macular degeneration, the optimum dosages of lutein, zeaxanthin, and the possible effects when combined with other nutrients.

## 5. Cardiovascular Disease

Despite the global decline in cardiovascular mortality, cardiovascular diseases remain the leading cause of morbidity and mortality, contributing to escalating health care cost [82]. Cardiovascular aging progresses over decades, influenced by risk factors such as tobacco use, poor physical activity and diet, resulting in hypertension, dyslipidemia (high triglycerides and lower HDL), elevated fasting blood glucose, and central obesity [83]. Cardiovascular disease is the major clinical problem in the older population, with 68% of adults 60–79 years having cardiovascular disease and this increases to 85% after the age of 80 years [84]. 

Good nutrition plays an important role in delaying the progression of cardiovascular disease [85,86]. The adverse effects of excess intakes of saturated and trans fats, cholesterol, added sugars, and salt in relation to cardiovascular disease progression has been relatively well-established whereas the effect of addressing inadequate essential nutrients is less well-known. Older adults are highly susceptible to undernutrition due to the various physiological and socioeconomic factors [87]. In contrast to overnutrition, the potential of addressing undernutrition to optimize cardiovascular health in older adults has received inadequate attention [88]. Evidence for nutrition in reducing the risk for cardiovascular aging mostly derives from epidemiological studies, whereas fewer interventions studies have been performed. The RCTs addressing cardiovascular disease generally have included, but not exclusively, older adults, not allowing generalizability of results to typical older adults. The authors have therefore focused on nutrition interventions addressing cardiovascular aging progress, not restricted to elderly. 

### 5.1. Cardiovascular Events

#### 5.1.1. Diets

Lifestyle changes, including dietary modifications, are recommended as part of the management strategy to improve lipid profiles and reduce the risk of cardiovascular disease [89,90,91]. The primary emphasis of dietary interventions has been on changing dietary macronutrient and salt composition. The effect of improving micronutrient-richness of the diet in cardiovascular disease control has been less-well studied. A diet rich in fruits, vegetables, wholegrains, legumes, nuts, fish, poultry, and low-fat dairy products, and limited consumption of red meat, saturated fat, and added sugar is advocated, mostly based on positive associations with cardiovascular health [89,90,91]. Dietary patterns that follow these principles include the Dietary Approaches to Stop Hypertension (DASH) diet, a diet rich in fiber, protein, magnesium, calcium, and potassium, and low in total and saturated fats, which has been shown to reduce low-density lipoprotein (LDL)-cholesterol levels [91], and the Mediterranean diet, which has been shown to reduce the risk for cardiovascular disease in both primary and secondary settings [92,93]. Regression of coronary artery atherosclerosis has been demonstrated with a program of intensive lifestyle changes that included a vegetarian diet, exercise, and smoking cessation [94]. In addition to dietary interventions, there has been research into the effects of individual nutrients. While the evidence for some of these is limited, several interesting findings have been published. 

#### 5.1.2. Vitamin D

Low vitamin D has been associated with cardiovascular disease in a number of studies [95]. Few studies have been targeting low vitamin D specifically in the older population. In one study with post-menopausal women randomized to Vitamin D3 2500 IU or placebo, daily for 4 months, vitamin D supplementation had no effect on endothelial function, arterial stiffness, or inflammation [96]. Results of a meta-analysis of RCT with older adult participants (≥60 years) suggested that vitamin D supplementation might protect against cardiac failure but not against MI or stroke [97]. The recent results of the VITAL trial indicate that daily supplementation of 2000 IU vitamin D did not reduce the occurrence of cardiovascular events in adults aged ≥50 years [98].

#### 5.1.3. B-Vitamins

**Figure 2:**
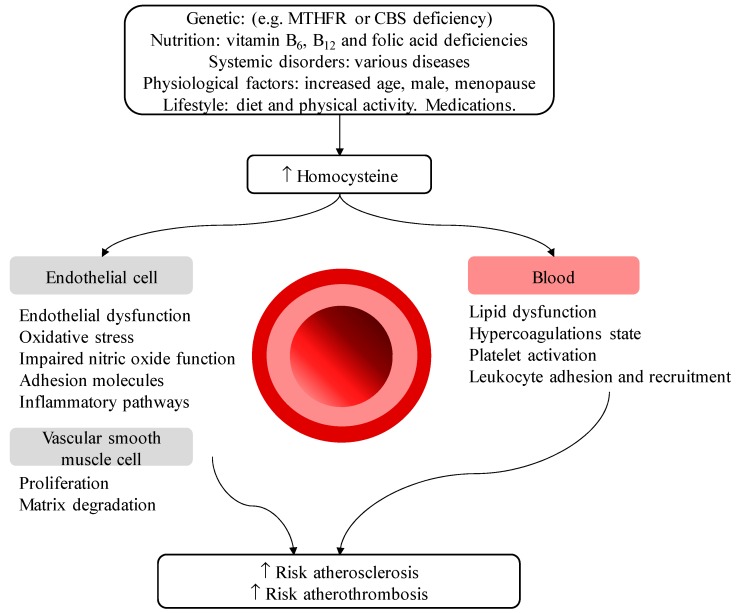
Risk factors and mechanisms for high homocysteine in cardiovascular disease. MTHFR: methylenetetrahydrofolate reductase, CBS: cystathionine beta-synthase.

B-vitamins have been the subject of substantial research because of their established effects on normalizing homocysteine levels, an important risk factor for cardiovascular disease. Figure 2 shows the risk factors including B-vitamin shortages and pathogenetic mechanisms for the effect of high homocysteine on cardiovascular disease.

Particularly the B-vitamins have been investigated for their potential cardiovascular benefits due to their established lowering effect on homocysteine levels, a marker for cardiovascular disease risk, including ischemic stroke. A meta-analysis of 19 RCTs of B vitamins (including folic acid, vitamin B6, vitamin B12, and B-complex vitamins) found significant reductions in homocysteine levels, however, no significant effect of vitamin B supplementation on rates of cardiovascular disease, coronary heart disease, myocardial infarction, cardiovascular death, or all-cause mortality whereas vitamin B reduced the risk of stroke by 12% [99]. Another meta-analysis of 26 RCTs found that folic acid supplementation significantly reduced the risk of stroke 7% [100]. 

There are various reasons for elevated blood homocysteine levels; most people have mild to moderately elevated serum homocysteine levels due to inadequate intake of folate, vitamin B_6_, or vitamin B_12_ from the diet, which is reversible when intake of these vitamins is increased. Another cause are genetic variants of methylenetetrahydrofolate reductase (MTHFR) and methionine synthase reductase (MTRR) that are associated with elevated homocysteine levels. Elevated homocysteine levels are a risk factor for developing blood clots in the vasculature and have been implicated in the pathogenesis of atherosclerosis and deep vein thrombosis [101]. Given that vitamin B supplementation is associated with normalization of elevated plasma homocysteine levels, many studies have investigated whether these vitamins may decrease the risk of cardiovascular diseases. Huang and colleagues undertook a meta-analysis (19 RCTs and 47,921 participants) evaluating the effects of B vitamin supplementation (search terms: folic acid, folate, vitamin B_6_, vitamin B_12_, and B vitamins) on plasma homocysteine levels and cardiovascular and all-cause mortality [99]. The overall relative risk of a clinical outcome, versus placebo, was 0.98 for cardiovascular disease, 0.98 for CHD, 0.97 for MI, 0.97 for cardiovascular death and 0.88 for stroke; and homocysteine levels were decreased in all RCTs. Thus, B vitamin supplementation had a significant protective effect for stroke, but not for any other cardiovascular risk. A more recent meta-analysis of folic acid supplementation (30 RCTs, 82,334 participants) estimated a 10% lower risk of stroke and a 4% lower risk of overall cardiovascular disease compared with controls [102]. The greatest benefit for cardiovascular disease was observed in individuals with lower plasma folate levels at baseline and without pre-existing cardiovascular disease (*p* = 0.006 for both). While patients with a cardiovascular disease history responded to B-vitamins with normalization of homocysteine levels, those with the MTHFR 677C > T genotype were less responsive and may have greater folate requirements than do their counterparts [103].

#### 5.1.4. Vitamin K

Vitamin K plays an important role in anticoagulation and may overcome the detrimental side effects associated with vitamin K antagonists such as warfarin. Vitamin K may also help to prevent vascular calcifications, especially in patients on warfarin [104]. 

#### 5.1.5. Omega-3 LCPUFA

Supplementation of omega-3 LCPUFA increased high-density lipoprotein (HDL) cholesterol concentration, improved vascular function, and lowered heart rate and blood pressure with DHA having a greater effect than EPA while both EPA and DHA inhibited platelet activity [105]. Dietary supplementation with omega-3 LCPUFAs can reduce plasma triglyceride levels by up to 45% [106,107], with the greatest effect seen in those with the highest baseline levels [106]. Omega-3 LCPUFAs also cause a modest increase in HDL-C levels, and although they also increase LDL-C levels, this is primarily an increase in large, less atherogenic, particles [106]. In addition to improving lipid profiles, omega-3 LCPUFAs reduce inflammation, lower blood pressure (blood pressure), and have beneficial effects on endothelial function and platelet aggregation, all of which could contribute to cardioprotective effects [106]. However, despite positive effects on intermediate markers, RCTs with omega-3 LCPUFAs have produced mixed results on cardiovascular morbidity and mortality [108,109]. It must be noted that these meta-analyses included both primary and secondary prevention studies, before and after occurrence of events, respectively. One recent meta-analysis of RCTs performed reported a significant reduction in cardiovascular risk only among higher risk populations, such as those with elevated triglyceride levels (relative risk: 0.84, 95% CI 0.72–0.98) or elevated LDL-cholesterol levels (relative risk 0.86, 95% CI 0.76–0.98) [108]. Another recent Cochrane meta-analysis of RCTs found that omega3 LCPUFAs reduced cardiovascular events in the main analysis (relative risk: 0.93, 95% CI 0.88–0.97), but the result was not maintained in sensitivity analyses [109]. The failure of some trials to show effects of omega-3 LCPUFA on cardiovascular disease was explained by an insufficiently high omega-3 LCPUFA dose and/or too high omega-3 LCPUFA baseline status to demonstrate effects [110]. RCTs evaluating the effects of omega-3 LCPUFAs on cardiovascular morbidity and mortality generally enrolled a broad range of ages while only few RCTs have focused specifically on older adults. The Alpha Omega Trial that included 60–80-year-olds with previous MI and at least 50% on medication found no significant effect of approximately 400 mg of omega-3 LCPUFA on cardiovascular events [111]. In the AREDS2 study, 1 g omega-3 LCPUFA given in addition to a standard Vitamin C, Vitamin E, beta-carotene, zinc oxide, and cupric oxide supplement for 6 months to participants between 50 and 85 years had no effect on cardiovascular outcomes [112]. The recent results of the VITAL trial showed that in adults aged ≥50 y daily consuming 840 mg of omega-3 LCPUFA lowered the risk of heart attack by 28%, of fatal heart attack by 50% without significant effect on stroke or cardiovascular deaths [98]. The most pronounced benefits on major cardiovascular event reduction were found in participants who reported low fish intake at baseline. A recent meta-analysis of RCTs found that omega-3 LCPUFA supplementation caused a small, but significant, reduction in heart rate (−2.23 bpm, 95% CI −3.07 to −1.40) [113], which is considered a risk factor for cardiovascular morbidity and mortality [114].

#### 5.1.6. Antioxidants

Inflammation and oxidative stress appear to be key drivers for a number of cardiovascular diseases and the metabolic syndrome [115,116]. Whereas observations studies suggest that antioxidant nutrient such as β-carotene and vitamin E are associated with lower cardiovascular disease, the data of RCTs on antioxidant supplements failed to confirm a significant benefit of antioxidants on atherosclerotic cardiovascular disease. For instance, supplementation with the antioxidant nutrients vitamin E, β-carotene, and vitamin C, had no significant effects on cardiovascular outcomes [117].

#### 5.1.7. Vitamin E

A key attribute of vitamin E (a combination of 8 distinct tocopherol/tocotrienol isoforms) is its antioxidant activity and, as a consequence, its ability to protect poly-unsaturated fatty acids (PUFAs), lipoproteins, and cell membranes from oxidative damage [118]. Vitamin E has been extensively investigated for its potential to prevent cardiovascular disease events. Nevertheless, RCTs with vitamin E had mixed results on various cardiovascular disease endpoints. In the Women’s Health Study, intake of 600 IU of vitamin E on alternate days in apparently healthy women non-significantly reduced the risk for cardiovascular events by 7% and significantly reduced the risk for cardiovascular death by 24% [119]. And among women ages 65 and older, vitamin E supplementation reduced the risk of major cardiac events by 26% [119]. Data from the same Women’s Health Study suggested that supplementation with vitamin E may reduce the risk of venous thromboembolism in women, particularly in those with a prior history or genetic predisposition [120]. RCTs that retrospectively analyzed the data for the effect of vitamin in E in subgroups of patients with this these genotypes sometimes showed that these patients are more responsive to vitamin E supplementation [121,122]. 

#### 5.1.8. Phenolics

Phenolic compounds are bioactive compounds found in plants, and there is evidence that some may be helpful for reducing cardiovascular risk factors [116]. Flavonoids are polyphenolic compounds found in fruits, vegetables, tea, and red wine [116]. Amongst the flavonoids, there is some evidence that flavonols (specifically quercetin) may be effective at reducing blood pressure in hypertensive patients; however, no effects on other cardiovascular disease risk markers such as endothelin, oxidative stress, or lipid profiles were found [116]. Although an early meta-analysis found that consumption of flavonols was associated with a lower rate of cardiovascular disease [123], a more recent meta-analysis and a systematic review do not support such an effect [116,124]. Amongst other phenolic compounds which might have beneficial cardiovascular effects, resveratrol is a stilbene found in grape skin, red wine, and peanuts [116]. A systematic review found that resveratrol was associated with reductions in total cholesterol, LDL-C, triglycerides and apolipoprotein B in a range of patients, including those with ischemic heart disease [116]. Resveratrol also reduced inflammatory and fibrinolytic biomarkers in patients with ischemic heart disease [116].

Nutrients have been investigated for their effect on cardiovascular disease progress and as such, outcomes. B-Vitamins reduced homocysteine levels, a risk factor of cardiovascular disease, without significant effects on cardiovascular disease events except for the reduced risk for stroke, which was also reduced by folic acid supplementation. Flavonoids and omega-3 LCPUFA also reduce cardiovascular disease risk factors although evidence on cardiovascular outcomes is mixed. Possible explanations include that patients enrolled in the RCTs were already at high risk of cardiovascular disease and on concomitant medications, with little opportunity for nutrition to reverse the progress. Individual nutrients like vitamin D, vitamin E and omega-3 LCPUFA on cardiovascular disease prevention have shown mixed effects. Nutrition interventions have focused mostly on primary prevention of cardiovascular aging in broad age groups and less on older adults. Recruited participants in the RCTs were often at high risk of cardiovascular risk factors or preexisting disease, a modest effect of in patients that already have heart disease or are at high risk of heart disease may be masked by effects of medication.

### 5.2. Hypertension

Hypertension is a major public health concern given its link to serious cardiovascular events such as stroke and ischemic heart disease, the leading causes of worldwide mortality [6]. It has been estimated that hypertension is responsible for approximately 40% of cardiovascular deaths. By the year 2025 almost 30% of the global population will be diagnosed with high blood pressure, with 25% of these cases occurring in developing countries [125]. Hypertension rises dramatically with aging due to longer exposure to age-associated alterations in vascular function and structure and cardiovascular risk factors [126].

Hypertension is a multifactorial disease with lifestyle factors such as physical activity, smoking and drinking habits, diet, bodyweight, and anxiety playing a predominant role. Management of these is the first step to achieving adequate blood pressure control. Indeed, it has been reported that two lifestyle modifications can help improve blood pressure control and decrease the number of cardiovascular outcomes [127]. 

#### 5.2.1. Diets

In the current healthcare environment, lifestyle changes involving a healthy diet and increased physical activity are considered pivotal in the management of hypertension. Diets with a high nutritional value, such as the traditional Mediterranean diet, DASH and the OmniHeart (a variation of DASH with increased levels of protein) diets, can be important steps on the path to weight loss, lowering blood pressure, and prevention of hypertension [125]. The benefits of the DASH diet on blood pressure were reported in a RCT with all participants receiving graded amounts of sodium (high, intermediate, low). There were dose-response decreases in systolic and diastolic blood pressures, and age-related increases in blood pressure were blunted [128]. Both the DASH diet and low sodium markedly decreased blood pressure, and the combined effect was even greater. Findings of the DASH study also provided additional support that the sodium-to-potassium ratio is stronger associated with blood pressure outcomes than either nutrient alone among prehypertensive and hypertensive adults combined. These findings were later confirmed by a systematic review showing that the sodium-to-potassium ratio appears to be more strongly associated with blood pressure outcomes than either nutrient alone in hypertensive adults [129]. 

In addition to dietary control there has been research into the effects of other nutrients, including vitamins, on blood pressure and hypertension. While the evidence for some of these is limited a number of interesting findings have been published. 

#### 5.2.2. Milk peptides

A meta-analysis of 14 RCTs involving 1306 European subjects found that the milk-derived lactotripeptides isoleucine-proline-proline and valine-proline-proline produced small and statistically significant reductions in mean systolic blood pressure and diastolic blood pressure [130]. The authors noted that a similar effect had been seen in Asian populations.

#### 5.2.3. Omega-3 LCPUFAs

The omega-3 LCPUFAs EPA and DHA found in oily fish and fish oils (including capsule preparations) have been associated with lower blood pressure levels. In a meta-analysis of 70 RCTs, EPA, and DHA reduced mean systolic blood pressure and mean diastolic blood pressure compared with placebo. The largest effect was in untreated hypertensive patients [131]. Likewise, in an earlier meta-analysis (36 trials), intake of fish oil (median dose 3.7 g/d) reduced both mean systolic and diastolic blood pressure. The antihypertensive effects of doses <0.5 g/d remains to be established [132].

#### 5.2.4. Vitamin C

In short-term studies, vitamin C supplementation reduced systolic and diastolic blood pressure. Long-term trials on the effects of vitamin C supplementation on BP and clinical events are needed longer-term trials assessing the effects of vitamin C supplementation on blood pressure and clinical events in patients with hypertension would seem to be worthwhile [133]

#### 5.2.5. Vitamin D

In a study involving 283 hypertensive patients, vitamin D3 (cholecalciferol) produced a modest but statistically significant reduction in systolic blood pressure compared with placebo after 3 months [134]. There was no significant effect on diastolic blood pressure.

#### 5.2.6. Flavonols

Flavanols have also been found to lower blood pressure, and there is some evidence suggesting that they improve endothelial function in patients with ischemic heart disease, but additional studies are needed [116].

The evidence for nutrients and blood pressure is convincing for lowering sodium and sodium-to-potassium ratio. Flavanols vitamin C and D may have modest significant effects on blood pressure lowering.

### 5.3. Diabetes

Type 2 diabetes has become a global health-related pandemic which is forecast to rise from 425 to almost 630 million by 2045 [135]. In developing countries, the forecasted increase is more alarming, particularly in regions which are more rapidly adopting a Western lifestyle. The direct financial burden on healthcare systems and society is huge, as are the indirect costs from loss of work attendance. Intensive lifestyle modification, e.g., personalized nutrition and physical activity programs, with the goal of improving glycaemia and losing excess body weight should be the mainstay of initial management in individuals with prediabetes [136]. 

#### 5.3.1. Vitamin D

Observational studies have highlighted a link between vitamin D deficiency and type 2 diabetes, as well as possible future cardiovascular events, whereas results from interventional studies have not been so conclusive [137]. A recent meta-analysis [137] including a total of 20 RCTs and 2703 participants, found that vitamin D supplementation was associated with elevated serum vitamin D levels and significantly decreased insulin resistance. Changes in other parameters such as fasting blood glucose and hemoglobin A1c (HbA1c) were relatively small and did not achieve statistical significance [137]. In a pilot study in 60 patients with co-existing type 2 diabetes and hypovitaminosis D, vitamin D improved vitamin D status and several parameters associated with glycemic control such as HbA1c, mean fasting plasma glucose, and mean post-prandial plasma glucose [138]. In addition, vitamin D in the study lowered LDL cholesterol levels, systolic blood pressure and diastolic blood pressure. 

#### 5.3.2. Vitamin E

Diabetes patients with the haptoglobin 2-2 genotype have elevated risk of cardiovascular disease events. The haptoglobin 2-2 genotype has inferior antioxidant properties as compared with other haptoglobin types resulting in elevated levels of oxidative stress, an atherogenic profile and an increased risk of cardiovascular disease events compared with other Hp genotypes [139]. The RCTs in diabetes patients that retrospectively analyzed the data for the effect of vitamin in E found that administration of vitamin E lowered the risk of cardiovascular disease events by 34% and cardiovascular-related mortality by 53% among patients with the haptoglobin 2-2 genotype [140]. 

#### 5.3.3. Omega-3 LCPUFA

Cohort studies have shown that in countries where fish consumption is high the prevalence of type 2 diabetes tends to be lower and this has been attributed to the presence of omega-3 LCPUFAs [141]. However, the findings have not been conclusive with respect to providing dietary guidance and a recent systematic meta-analysis sought to provide more definitive evidence by analyzing different dosage/compositions of omega-3 LCPUFA supplementation [141]. In total, 20 RCTs recruited 1209 patients with type 2 diabetes. Overall, omega-3 LCPUFA supplementation resulted in a reduction in triglycerides with the best response with high doses for a longer duration; however, no significant changes in total cholesterol, fasting plasma glucose, post-prandial plasma glucose, HbA1c, insulin, or body mass was noted with this regimen. Interestingly, products with a relatively high ratio of EPA to DHA exhibited an increasing tendency to decrease HbA1c, insulin, total cholesterol, total triglycerides, and body mass. These findings will be helpful for clinicians and nutritionists who manage patients with diabetes to provide dietary guidance [141].

#### 5.3.4. Vitamin K

To assess whether vitamin K is a risk factor for the development of type 2 diabetes mellitus, Beulens and colleagues analyzed a cohort of 38,094 Dutch men and women over a 10-year period [142]. The study showed that both vitamin K_1_ and vitamin K_2_ intake were associated with a reduced risk of type 2 diabetes mellitus. For vitamin K_1_ the risk reduction occurred at the higher levels of intake, whereas for vitamin K_2_ a linear inverse association was established. In older men with diabetes receiving vitamin K_1_ supplementation for 36 months, vitamin K_1_ significantly improved insulin sensitivity [143].

#### 5.3.5. Chromium

Chromium plays a role in insulin metabolism by activating oligopeptide low-molecular-weight chromium (LMWCr)-binding substance and activating insulin-dependent kinase activity. A meta-analysis of the efficacy of chromium supplementation suggest that there is available evidence for chromium on glycemic control in patients with diabetes [144].

Studies in diabetes patients showed that vitamin D supplementation can improve serum vitamin D levels and significantly decrease insulin resistance. Currently, a large multicenter RCT is ongoing in the US (Vitamin D and Type 2 Diabetes Study; D2d), hypothesizing that vitamin D will enhance insulin production, glucose processing and glycemic profiles. Subgroup analyses show that vitamin E may be promising in reducing the rate of cardiovascular events among diabetes patients with haptoglobin 2-2 genotype who are at increased risk of cardiovascular events. The evidence for omega3 LCPUFA supplementation on fasting plasma glucose or HbA1C is less conclusive but omega-3 LCPUFA have promising effects for reduction of triglycerides. The evidence for chromium in glycemic control is emerging.

## 6. Conclusions

Inadequate or even deficient nutrient intake and status is still widely prevalent at global level and, although generally underacknowledged, is a main risk factor for NCDs [20]. Nutrient surveys indicate that the aging population is at particular risk for poor nutrient intake and status, which may result in increased risk for chronic fatigue, and cardiovascular, cognitive, and neuromuscular disorders in older adults. The present paper reviews the evidence for the role of various nutrients in modifying the risk of development of NCDs throughout aging. 

Inadequate vitamin D, calcium and vitamin K intake and status are generally reported in the aging population and have been associated with musculoskeletal disorders, such as increased bone fracture risks. Increased vitamin D in combination with increased calcium and possibly also vitamin K may reduce the risk for hip fractures, thus beneficially impacting musculoskeletal health.

Inadequate B vitamins intake and status, in particular folic acid, vitamins B_6_ and B_12_, have been associated with age-related cognitive decline, while supplementation has been reported to improve cognitive performance. Similarly, evidence has been reported for vitamin C, D, and E, as well as omega-3 LCPUFAs (e.g., DHA) to slow down dementia progression.

Increased intake of lutein and zeaxanthin has been demonstrated to improve macular pigment optical density measures, a marker of age-related macular degeneration. 

Various nutrients have been reported to play a role in reducing the risk for ischemic heart disease, stroke, myocardial infarction, heart failure, hypertension, and diabetes with varying levels of effect size and evidence. B-Vitamins reduced homocysteine levels and reduced the risk for stroke. Some but not all studies reported that higher omega-3 LCPUFAs intakes resulted in reduced risk of cardiovascular events; most pronounced effects being shown in subjects with low intake or status. Vitamin C and D may reduce hypertension, omega-3 LCPUFAs may have positive effect on blood lipid profiles, and omega-3 LCPUFAs, vitamin D, and chromium may reduce diabetes risk factors.

Most pronounced benefits of nutrient interventions were sometimes found in subgroups which had low baseline intake or status of the nutrient. Genetic factors can affect the status of certain nutrients, as well as contribute to increased risk for NCDs and raise the needs for certain nutrients [139,145]. Targeted supplementation with nutrients of concern to genetically predisposed subgroups has been shown to confer benefits as shown by some examples in this review. More research is needed to unravel the benefits of optimizing nutrition where it is needed, for instance by targeting those at increased risk for NCDs linked to low nutrition status or genetic profile.

Due to a growing aging global population, related NCDs including musculoskeletal disorders, dementia, loss of vision, and cardiovascular diseases will place an increasing burden on health systems and costs. Adequate nutrient status may help to improve health and wellbeing in older populations and slow the progression of NCDs. Implementing a long-term preventative strategy to promote healthy aging and break down the barriers to adequate nutrition for older adults could result in significant healthcare cost savings. Nutrition is increasingly acknowledged and integrated into public health policies and programs to manage healthy aging. Promoting nutrient-rich diets and adequate nutrient intakes for healthy aging should be considered part of an integral approach to address NCDs in health policies. There is a need for public and/or private partnerships where governments, health authorities, academics, and the food sector jointly promote the benefits of healthy nutrient-rich diets and lifestyle to manage NCDs. 

In conclusion, data indicate that inadequate nutrient intake and status is common in older aged adults and represents a risk for the development of NCDs during aging. Studies for the aging population have demonstrated that optimizing nutrition can reduce the risk and progress of NCDs. Although the scientific evidence is not conclusive for all health benefits, it should not prevent health authorities from promoting balanced and adequate nutrient intakes as integral part of nutrition strategies to reduce the burden of NCDs associated with inadequate nutrition.

## Figures and Tables

**Table 1:** Percentage of adults with nutrient intakes meeting the estimated average requirement (EAR) or adequate intake (AI) or exceeding the maximum reference value (MRV) [12].

% Meeting EAR or AI	EAR or AI	Denmark *n* = 2025 people	Czech Republic *n* = 1869 people	Italy *n* = 2831 people	France *n* = 2624 people
Protein, g/d	0.66 g/kg BW	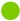 84%	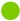 88%	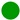 99%	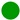 98%
MUFA, E%	10–20 E%	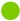 69%	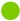 92%	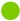 75%	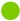 77%
Dietary fiber, g/d	25	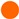 19%	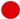 4%	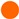 12%	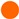 9%
Calcium, mg/d	750	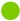 70%	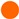 31%	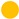 43%	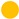 62%
Iron, mg/d	M: 6; F: 7	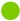 92%	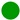 96%	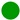 98%	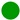 98%
Potassium, mg/d	3500	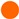 31%	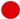 4%	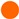 19%	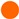 18%
Magnesium, mg/d	M: 350; F: 300	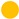 46%	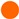 25%	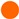 20%	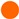 23%
Zinc, mg/d	M: 7.5; F: 6.2	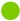 90%	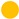 48%	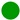 97%	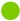 91%
Vitamin A, µg RE/d	M: 570; F490	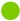 77%	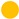 38%	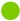 66%	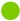 77%
Vitamin C, mg/d	M: 90; F: 80	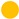 50%	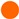 35%	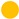 62%	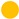 44%
Vitamin E, mg/d	M: 13; F: 11	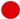 5%	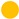 44%	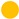 47%	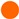 34%
Vitamin D, µg/d	15	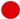 3%	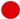 1%	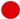 1%	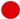 1%
Vitamin B_1_, mg/d	0.6	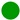 97%	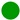 98%	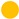 47%	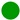 100%
Vitamin B_2_, mg/d	M: 1.1; F: 0.9	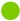 80%	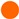 35%	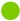 84%	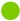 92%
Vitamin B_12_, µg/d	4	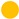 55%	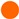 36%	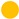 52%	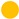 50%
Folate, µg DFE/d	250	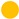 59%	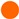 24%	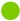 77%	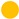 51%
**% exceeding MRV**		**MRV**			
SFA, E%	<10 E%	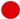 86%	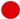 80%	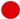 62%	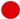 91%
Added sugar, E%	<10 E%	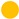 32%	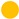 21%	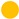 24%	
Sodium, mg/d	<2400 mg/d	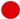 80%	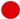 98%	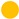 13%	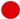 85%

RE: retinol equivalents, DFE: dietary folate equivalents, E%: energy percentage, MUFA: mono-unsaturated fatty acids, SFA: saturated fatty acids. The red, orange, yellow, light green and dark green signals, respectively, represent ≤5%, 6–35%, 36–65%, 66–95%, and ≥96% of people meeting the EAR.

**Table 2:** Critical nutrients in older adults [18].

Micronutrient	Challenges, Clinical Signs, and Symptoms in Older Adults
Vitamin B12 (cobalamin)	Deficiencies common in older adults, often underdiagnosed. Role in reducing elevated homocysteine, a cardiovascular risk factor. Absorption decreases mainly due to high prevalence of age-related atrophic gastritis. Among the common causes of anaemia in older adults, leading to weakness and fatigue. Low status increases the risk for cardiovascular disease and cognitive impairment.
Folate	Deficiencies common in older adults. Role in reducing elevated homocysteine, a cardiovascular risk factor. Closely related to vitamin B12 and B6. Among the common causes of anaemia in older adults, leading to weakness and fatigue. Deficiencies linked to depression and dementia.
Vitamin B6	Deficiencies common in older adults. Role in reducing elevated homocysteine, a cardiovascular risk factor. Closely related to vitamin B12 and folate.
Thiamine (vitamin B1)	Deficiencies common in older adults, often underdiagnosed. Risk factor for heart failure, peripheral neuropathy, and encephalopathy.
Calcium	Deficiencies common in senior women. Mean intake decreases with age, probably related to general change in diet. Associated with low bone mass, rapid bone loss, and high fracture rates.
Vitamin D	Older adults are less exposed to sun and have diminished ability of the skin to synthesize vitamin and the liver and kidney to hydrolyze vitamin D with age. Deficiency is a risk factor low bone mass, rapid bone loss, high fracture rates, and muscle weakness.
Vitamin C	Prevalence of inadequate intake is very high among adults. May help elderly maintain immune cells and function. Smoking increases need.
Iron	Women’s iron requirements decrease after the menopause. Deficiencies are mainly seen among hospitalized, institutionalized, or chronically ill older adults. Among the common causes of anaemia in older adults, leading to weakness and fatigue.
Zinc	Deficiency is common in the elderly. Risk factor for immune deficiency and susceptibility to infection in the elderly.
Selenium	Deficiency deficiency may increase risk of diseases of aging such as cardiovascular disease, reduced immune response, and cognitive decline.
Magnesium	Often deficient in older adults. Maintains muscle integrity and function.
